# Mass-Spectrometry Based Proteome Comparison of Extracellular Vesicle Isolation Methods: Comparison of ME-kit, Size-Exclusion Chromatography, and High-Speed Centrifugation

**DOI:** 10.3390/biomedicines8080246

**Published:** 2020-07-25

**Authors:** Anders Askeland, Anne Borup, Ole Østergaard, Jesper V. Olsen, Sigrid M. Lund, Gunna Christiansen, Søren R. Kristensen, Niels H. H. Heegaard, Shona Pedersen

**Affiliations:** 1 Department of Clinical Biochemistry, Aalborg University Hospital, DK-9000 Aalborg, Denmark; anneborup@clin.au.dk (A.B.); sigridlarsen@hotmail.com (S.M.L.); srk@rn.dk (S.R.K.); 2 Department of Autoimmunology and Biomarkers, Statens Serum Institut, DK-2300 Copenhagen, Denmark; ole.ostergaard@cpr.ku.dk (O.Ø.); NHE@ssi.dk (N.H.H.H.); 3 Novo Nordisk Foundation Center for Protein Research, Faculty of Health and Medical Sciences, University of Copenhagen, DK-2200 Copenhagen, Denmark; jesper.olsen@cpr.ku.dk; 4 Department of Medical Microbiology and Immunology, University of Aarhus, DK-8000 Aarhus, Denmark; gunna@loke.dk; 5 Department of Clinical Medicine, Aalborg University, DK-9000 Aalborg, Denmark; 6 Department of Clinical Biochemistry and Pharmacology, Odense University Hospital, University of Southern Denmark, DK-5000 Odense, Denmark

**Keywords:** Extracellular vesicles, human plasma, EV isolation, high-speed centrifugation, size exclusion chromatography, ME kit, peptide affinity, mass spectrometry, proteomics, proteome

## Abstract

Extracellular vesicles (EVs) are small membrane-enclosed particles released by cells under various conditions specific to cells’ biological states. Hence, mass-spectrometry (MS) based proteome analysis of EVs in plasma has gained much attention as a method to discover novel protein biomarkers. MS analysis of EVs in plasma is challenging and EV isolation is usually necessary. Therefore, we compared differences in abundance, subtypes, and contamination for EVs isolated by high-speed centrifugation, size exclusion chromatography (SEC), and peptide-affinity precipitation (PAP/ME kit) for subsequent MS-based proteome analysis. Successful EV isolation was evaluated by nanoparticle-tracking analysis, immunoblotting, and transmission electron microscopy, while EV abundance, EV subtypes, and contamination was evaluated by label-free tandem MS. High-speed centrifugation and SEC isolates showed high EV abundance at the expense of contamination by non-EV proteins and lipoproteins, respectively. These two methods also resulted in EVs of a similar type, however, with smaller EVs in SEC isolates. PAP isolates had a relatively low EV abundance and high contamination. We consider high-speed centrifugation and SEC suitable as EV isolation for MS biomarker studies, where the choice between the two should depend on the scientific questions and whether the focus is on larger or smaller EVs or a combination of both.

## 1. Introduction

Blood plasma is a minimally-invasive source for discovering protein biomarkers for disease diagnostics, screening, monitoring, and evaluation of therapeutic responses [1,2]. In this context, mass spectrometry (MS) based proteome analysis is an attractive analytical technique. MS can analyse and quantify thousands of proteins in an unbiased way covering 5–6 orders of magnitude in protein abundances. However, as plasma proteins span more than 12 orders of magnitude, MS is limited to analysis of only the most abundant proteins in non-processed plasma [3].

Focusing the analysis on extracellular vesicles (EVs) contained within plasma is a new diagnostic possibility, enabling the analysis of protein biomarkers despite the complexity of plasma. EVs are a heterogeneous group of membrane-enclosed particles that are released from cells into the intercellular space and distributed throughout circulating biofluids, including blood. EVs have a size between 30 and 1000 nm and include two main subtypes, namely exosomes (30–150 nm) and microvesicles (MVs; 100–1000 nm) [4]. A similar size and morphology make the differentiation between EV subtypes difficult [5]. Functionally, EVs are involved in intercellular communication via the transfer of proteins, DNA, RNA, and bioactive lipids [6]. Furthermore, since EVs are released from specific cells they may carry disease-specific proteins [7]. Combined, these attributes make EV characterization difficult, but also an excellent source for discovering biomarkers.

EVs account for only a small fraction of the total content of plasma and hence should be isolated before MS analysis. EV isolation methods can be classified as (1) centrifugation based, (2) filtration techniques, (3) chemical precipitation, or (4) affinity precipitation [8,9]. Centrifugation separates EVs from other plasma components based on the relatively higher density of EVs, enabling larger EVs to be pelleted at ~20,000× *g* and smaller EVs to be pelleted at ~100,000× *g* [10,11]. EV isolation by filtration is primarily performed using size exclusion chromatography (SEC), where EVs are separated from soluble plasma proteins due to the larger size of EVs [12]. Chemical precipitation works by polymer co-precipitation, where polymers interact with EVs and enable EV extraction. Polymers are usually co-isolated during chemical precipitation, which in turn makes the isolates incompatible with MS analysis [9,13]. Affinity precipitation isolates EVs by specifically targeting EV epitopes [8]. Historically, affinity capture methods have produced highly specific EV isolates, however, more recently developed methods now enable the isolation of broader EV populations. One such method is the ME Kit by New England Peptide which uses peptide affinity precipitation (PAP), where a peptide fragment binds to heat shock protein 70 (HSP70) expressed on EVs and enables them to be isolated by subsequent centrifugation [14,15].

The isolation of EVs from plasma is challenging due to its complexity, where isolation typically results in the co-isolation of confounding and highly abundant non-EV proteins, protein aggregates, and lipoproteins which can suppress other interesting proteins [13]. Thus, for an EV isolate to be applicable for MS-based biomarker discovery, the isolation method must ensure adequate EV abundance and low contamination [16]. Two such highly abundant contaminants in plasma are albumin and lipoproteins. Albumin is common in centrifugation-based isolates, due to an inability of centrifugation to remove highly abundant plasma proteins completely. Pellet washing, where centrifugation is repeated on a pellet with a fresh buffer can mitigate this at the cost of a lowered EV yield [10]. Lipoproteins are commonly found in SEC isolates due to the overlapping size ranges between certain lipoproteins and EVs [17,18].

In the present work, we evaluated three EV isolation methods on plasma for their applicability in clinical proteomics and biomarker discovery. The isolation methods compared were high-speed centrifugation [11], SEC [16], and PAP [14]. We evaluated differences in EV abundance, EV subtypes, and sample contamination based on proteome characterization by MS.

## 2. Experimental Section

### 2.1. Study Population

Subjects included in this study were recruited at Aalborg University Hospital, Denmark. Subjects included three healthy volunteers: two males and one female. Samples were collected following local and regional ethical guidelines after participants had given written consent (ethical approval identifier: *N-20130075*; approval date: 16 March 2016). All participants were requested to o vernight fasting before sample collection, ensuring no undesirable increases in lipoproteins in the blood [19].

### 2.2. Pre-Analytical Procedures

Venous blood was obtained by antecubital venepuncture using a 21-gauge needle and collected into 6 mL BD Vacutainer^®^ coagulation sodium citrate (3.2%, 105 mM) tubes (BD Bioscience, New Jersey, USA, Cat#366575). The use of a large 21-gauge needle limits potential EV release caused by shear forces and cell activation. The first tube was discarded to minimize EVs originating from contact activation of coagulation [19]. To obtain platelet-poor plasma (PPP), whole blood was centrifuged 2 × 2500× *g* for 15 min at room temperature (Multifuge 3 S-R, Heraeus, Hanau, Germany), collecting the supernatant after each spin. PPP was cryo-preserved in 1 mL aliquots by snap-freezing in liquid nitrogen. For all samples, the time spent from collection until freezing did not exceed one hour. The frozen PPP was stored at −80 °C until analysis.

### 2.3. Extracellular Vesicle Isolation

Before EV isolation, the PPP was thawed by incubation in an ice-water mixture for one hour. This minimizes EV stress and potential rupture. EV isolation was performed in duplicates to evaluate the reproducibility of the protocols. Furthermore, to enable comparison of EV abundance independent of output volume, all EV isolates were corrected to a final volume of 50 µL isolate per 1 mL starting volume.

High-speed centrifugation was performed as previously described [10]. In brief, 1000 µL PPP was centrifuged in a fixed angle rotor at 18,890× *g* for 30 min at room temperature. After centrifugation, the supernatant was discarded and the pellet was washed in phosphate-buffered saline (PBS)-citrate buffer (154 mM NaCl, 1.24 mM Na_2_HPO_4_·2H_2_O, 0.205 mM NaH_2_PO_4_·2H_2_O, 0.32% *w*/*v* trisodium citrate). This procedure was conducted four additional times, resulting in a total of five centrifugations and four washes. After the final centrifugation, the supernatant was removed, and the pellet was resuspended in 50 μL PBS-citrate buffer.

SEC was conducted using commercially available “qEV Original” SEC columns (Izon Science Ltd., Oxford, England, Cat. #SP1) according to the manufacturer’s instructions. In brief, columns were loaded with 500 µL PPP. The SEC fractions were collected at once after sample loading and included 3 mL waste (fraction 1–6) and a 1.5 mL EV isolate (fraction 7–9). Both waste and EV isolate were collected in 1.5 mL fractions to minimize collection biases caused by excessive vial changes. For each sample, EVs were collected from two SEC columns, pooled, and corrected to a final volume of 50 μL by ultrafiltration using 100 kDa MWCO spin filters (Amicon-Ultra-0.5 mL, Merck Millipore, MA, USA, Cat. #UFC510096).

PAP isolation was performed using the ME kit (New England Peptide, MA, USA, Cat. #ME-020p-kit), where EVs are precipitated through the binding of a peptide (Vn96) to HSP70 expressed on EV surfaces [14,15]. The procedure was conducted following the manufacturer’s instructions (SOP version: 2-0316). Briefly explained, 1000 µL PPP was mixed with 1× PlasME buffer and Vn96 peptide, incubated for 30–45 min on a tube rotator, and centrifuged using a fixed angle rotor at 17,000× *g* for 15 min at room temperature. After centrifugation, the supernatant was removed, and the pellet washed in 500 µL PBS-citrate. This procedure was repeated three times using a shortened 10 min centrifugation. After the last centrifugation, the supernatant was removed, and the pellet was resuspended in 50 μL PBS-citrate.

### 2.4. Nanoparticle Tracking Analysis

Particle concentration and mode size were determined by nanoparticle tracking analysis (NTA) using a NanoSight LM10-HS (Malvern Instruments Ltd., Malvern, UK) equipped with a blue laser (405 nm) and Luca-DL EMCCD camera (Andor Technology, Belfast, UK). For analysis, particles were tracked for 5 × 30 s with the following settings: camera level 11, detection threshold 3, blur at auto, and sample viscosity set to water. EV concentration and size distributions were obtained by processing raw data by the NTA Nanosight software version 3.0 (Malvern, Worcestershire, UK). Standard 0.1 µM silica beads (Polysciences Inc, Warrington, PA, USA, Cat. #24041-10) were routinely measured as quality control.

### 2.5. Transmission Electron Microscopy with Immune-Gold Labelling

EVs were phenotypically and structurally characterized by transmission electron microscopy (TEM) with and without immuno-gold labelling against CD9. Procedurally, one drop from the EV isolate was placed on a glow discharged 400-mesh copper grid, washed in one drop PBS for 3 × 30 s, and blocked for five minutes in one drop 0.5% ovalbumin in PBS. After blockage, grids were incubated in one drop of primary antibody solution of mouse anti-human CD9 (BD Pharmingen™, San José, CA, USA, Cat. #555370; diluted 1:50 in 0.5% ovalbumin in PBS) for 30 min at 37 °C, washed in PBS, and incubated with 10 nm gold-coupled (British Biocell, Cardiff, UK) secondary goat anti-mouse antibody (diluted 1:25 in 0.5% ovalbumin in PBS) for 30 min at 37 °C. Grids were then washed (3 × 5 min in PBS + 3 × 5 min in 1% cold fish gelatin in PBS + 3 × 30 min in PBS), stained in one drop 1% (*w*/*v*) phosphotungstic acid (pH 7.0) and blotted dry. Grid visualization was performed using a JEM 1010 electron microscope (JEOL Ltd., Tokyo, Japan) with an electron accelerating voltage of 60 keV. Digital images were captured using a bottom-mounted KeenView-G2 CCD camera (Kamiina-gun, Nagano, Japan) and size was estimated using a 2160 lines/mm grid size replica. The EV isolates obtained by PAP were incubated with 2 M NaCl to separate the Vn96 peptide from the isolated EVs following the manufacturer’s instructions (SOP version: 2-0316, New England Peptide, Gardner, MA, USA) prior to TEM.

### 2.6. Immunoblotting

Immunoblotting was used to confirm EV isolation and compare lipoprotein contamination. Successful EVs isolation was determined by blotting against CD9 using mouse anti-human CD9 (diluted 1:500; BD Pharmingen™, San José, CA, USA, Cat. #555370) while lipoprotein contamination was assessed by blotting against mouse anti-human apolipoprotein B (Apolipoprotein B monoclonal antibody; ApoB-48/100; diluted 1:1000; Thermo Fisher Scientific, Waltham, MA, USA, Cat. #MIA1609). Briefly, 2 µg protein was loaded and separated by SDS-PAGE on Mini-PROTEAN TGX gels (Bio-Rad Laboratories, Hercules, CA, USA, Cat. #4561083) under non-reducing conditions. For immunoblotting, proteins were transferred to an Amersham Hybond P 0.2 μm polyvinylidene fluoride (PVDF) blotting membrane (GE Healthcare, Little Chalfont, UK, Cat. #10600021), blocked with 5% *w*/*v* skim milk, 0.1% *v*/*v* Tween-20 in PBS, and incubated overnight at 4 °C with primary antibodies in 2.5% *w*/*v* skim milk, 0.1% *w*/*v* Tween-20 in PBS. After incubation, the membrane was washed with PBST (0.1% *v*/*v* Tween-20 in PBS; 3 × 10 min) and incubated two hours at room temperature with goat anti-mouse secondary antibody (diluted 1:30,000; DAKO, Glostrup, Denmark, Cat. #P0447) and Precision Protein™ StrepTactin-Horseradish Peroxidase (diluted 1:500; Bio-Rad Laboratories, Hercules, CA, USA, Cat. #1610380). For visualization, Pierce ECL Prime (GE Healthcare, Little Chalfont, UK, Cat. #RPN2232) was added to generate a chemiluminescent signal. Blot imaging was performed on a PXI 4 Touch (Syngene Ltd., Cambridge, UK) apparatus using GeneSys version 1.5.4.0. Immunoblotting for centrifugation isolates against CD9 was performed on a separate gel due to incorrect sample loading during the first blot.

### 2.7. Tandem Mass Spectrometry

For MS analysis, EV isolates were prepared by protein precipitation using trichloroacetic acid/acetone by applying the first steps from a 2-D Clean-up Kit (Bio-Rad Laboratories, Hercules, CA, USA, Cat. # 163-2130). After precipitation, the proteins were re-solubilized in 10 µL 8 M urea, 50 mM NH_4_HCO_3_, reduced and alkylated using dithiothreitol (10 mM final concentration) and iodoacetamide (50 mM final concentration), and digested three hours at room temperature using endo-Lys C (Waco Pure Chemical Industries; 0.5 µg endo-Lys C/50 µL EV isolate). Then samples were diluted to 2 M urea using 50 mM NH_4_HCO_3_ and digestion was continued overnight by the addition of sequencing grade modified trypsin (Promega, Madison, WI, USA, Cat. #V5111; 1 µg trypsin/50 µL EV isolate).

EVs isolated by PAP were mixed with 4× sample buffer (Invitrogen, Carlsbad, CA, USA, Cat. #NP0007) and run on a 4–12% Bis-Tris NuPAGE gel (Life Technologies, Carlsbad CA, USA, Cat. #NP0321) until the dye front had migrated halfway through the gel electrophoresis was then stopped, and the gel stained with Instant Blue (Expedeon, Cambridgeshire, UK, Cat. #ISB1L). After staining the whole lane above the Vn96 peptide band was excised, cut into 1 mm gel-pieces and transferred to an Eppendorf-tube before destaining and overnight in-gel digestion by a standard protocol [20].

Samples were then acidified by addition of formic acid (2% *v*/*v* final concentration) and desalted on pre-equilibrated homemade StageTip columns containing C18 Empore Disks (3 M, Minneapolis, MN) [21] by washing with 20 μL 0.1% formic acid followed by peptide elution with 20 μL 50% acetonitrile, 0.1% formic acid into a 0.65 mL Eppendorf tube. The eluted peptides were vacuum concentrated until almost complete dryness and re-dissolved in 10 μL 5% *v*/*v* acetonitrile, 0.1% trifluoroacetic acid. The peptide concentration was estimated by absorbance at 280 nm using a NanoDrop 2000 (Thermo Fisher Scientific, Waltham, MA, USA).

Liquid chromatography coupled tandem mass spectrometry (LC-MS/MS) was performed by loading 5 µL (200 ng) desalted peptides directly onto a homemade 15 cm C18 column (1.9 μm Reprosil-Pur C18 beads, Dr. Maisch, Ammerbuch, Germany) using an EASY nLC-1200 system (Thermo Fisher Scientific, San José, CA, USA). The column was maintained at constant temperature (40 °C) using an integrated column oven (PRSO-V1, Sonation GmbH, Biberach, Germany) and interfaced online with a Q-Exactive HF mass spectrometer (Thermo Fisher Scientific, San José, CA, USA). Peptides were eluted from the column using a binary gradient (Solvent A: 0.1% *v*/*v* formic acid in H_2_O, solvent B: 80% *v*/*v* acetonitrile, 0.1% *v*/*v* formic acid in H_2_O) that went from 5% solvent B (*t* = 0 min) to 29% solvent B (*t* = 40 min), 55% solvent B (*t* = 60 min) and 95% solvent B (*t* = 62 min). After 10 min (*t* = 72 min) the data recording was stopped, and the gradient went back to 5% solvent B (*t* = 75 min) and the column was re-equilibrated 2 min in 5% solvent B.

MS data were acquired by recording full scan spectra (300–1500 mass/charge) in profile mode at resolution 120,000 (at *m*/*z* 200). MS/MS data were recorded in parallel in centroid mode using data-dependent fragmentation of the 12 most abundant ions (charge state two or higher) by higher-energy collisional dissociation using normalized collision energy set to 28 and dynamic exclusion set to 30 s. Spray voltage was set to 2 kV, heated capillary temperature to 275 °C, and the full scan and fragment scan target values were set to 3e6 (max injection time 15 ms) and 1e5 (max injection time 45 ms), respectively.

### 2.8. Protein Identification and Quantification from MS

Raw-files were analysed using MaxQuant (version 1.5.3.8, Max Planck Institute of Biochemistry, Martinsried, Germany) [22,23] for label-free peptide quantitation by MS1-intensity and peptides (proteins) were identified using the built-in Andromeda search engine [24]. The MaxQuant analysis was performed with the following settings; Digestion enzyme: Trypsin with maximum 2 missed cleavage sites. Precursor mass tolerance was 4.5 ppm and fragment mass tolerance was 20 ppm. Variable modifications: Oxidation (M) and acetyl (protein N-terminal). Fixed modifications: Carbamidomethyl (C). Multiplicity: 1 (no isotope labelling). Peptide false-discovery rate (FDR) 1%, protein FDR 1%, minimum peptides: 1. Match between runs was on (match window 0.7 min, alignment window 20 min). All other settings were default.

Proteins were identified by peptide-spectral matching searching against FASTA files containing the complete human proteome including 70,902 protein sequences (downloaded 2016-11-08 from UniProt [25]) and against a file containing common contaminants often encountered in LC-MS/MS-based proteome analysis supplied with MaxQuant. In addition, MaxQuant was set to automatically generate a decoy FASTA-file with the reversed sequences of the above two files to estimate false positive peptide spectral matches using a target-decoy strategy [26]. Identifications flagged by MaxQuant as CON (contaminants), REV (reverse hits from the decoy sequences), and Only-By-Site were removed from the protein tables before further analysis. For quantitative comparison of protein abundances between isolation methods, relative intensity-based absolute quantification values (riBAQ values [27,28]) were used (Table S1). riBAQ values were used as the assumption for LFQ quantification (that most proteins are unchanged between samples) could not be met. riBAQ is a locally normalized estimate of protein abundances calculated from iBAQ value as follows:

(1) $$riBAQ_{Protein\;i\;}=\frac{iBAQ_{Protein\;i}}{\sum_{j\;\in Proteins}iBAQ_{Protein\;i\;}}$$

Furthermore, to estimate relative EV abundance and relative differences in EV subtypes, the protein abundance of commonly expressed EV associated proteins specific and common for small and large EVs as previously defined by Kowal et al. [5] was used. For estimates of relative EV abundance a combined value of protein abundance for small, large, and common EVs were used, while the relative abundance for small and large EVs were based only on the representative protein abundances (Tables S1 and S2):

(2) $$\underset{j\;\in \;\mathrm{EV}\;\mathrm{recovery}}{\sum }{\mathrm{riBAQ}}_{\mathrm{Protein}\;j}=\underset{j\;\in \;\mathrm{Small}\;\mathrm{EV}\;\mathrm{markers}}{\sum }{\mathrm{riBAQ}}_{\mathrm{Protein}\;j}+\underset{j\;\in \;\mathrm{Large}\;\mathrm{EV}\;\mathrm{markers}}{\sum }{\mathrm{riBAQ}}_{\mathrm{Protein}\;j}+\underset{j\;\in \;\mathrm{Common}\;\mathrm{EV}\;\mathrm{markers}}{\sum }{\mathrm{riBAQ}}_{\mathrm{Protein}\;j}$$

Lastly, to calculate the relative sample contamination, we compared the relative protein abundances of lipoproteins, serum albumin, and non-EV proteins (Tables S1 and S2). For comparison of non-EV proteins, we adapted a technique previously described by Clark et al. [29] that used eight surrogate non-EV proteins based on their absence from the Exocarta database [30]. Here, we take this approach further and include all proteins from our dataset absent from the Exocarta database as surrogate non-EV protein markers, in total 66 proteins.

### 2.9. Statistical Analysis

Raw protein data from MaxQuant was analysed using Perseus (version 1.6.0.7, Max Planck Institute of Biochemistry, Martinsried, Germany) [31]. Data from NTA and figures were analysed and generated in R version 4.0.2 (https://www.R-project.org/). Processed and raw MS data, proteins used as EV and contamination markers, NTA data, unedited figures, reproducible R code, and Perseus analysis files are supplied as supplementary material (Table S1–S3; File S1–S6). For quantitative comparison, proteins were categorized and grouped into EV specific proteins (to compare relative EV abundance), EV subtype-specific proteins [5] (to compare differences in small and large EVs), and contaminants (to compare the amount of lipoproteins, serum albumin, and non-EV proteins), (Table S2). For statistical inference testing, the isolation methods were compared using independent samples t-tests. Comparison of EV abundance, EV subtypes, and EV contamination was done using FDR correction (settings; permutation-based FDR: 5%; S0: 0). Correlation between technical replicates was compared by calculating the coefficient of determination (*R^2^*) between technical replicates and biological samples. Statistical significance is illustrated by * (*p*-values < 0.05), ** (*p*-values < 0.01), and *** (*p*-values < 0.001).

## 3. Results

**Figure 1:**
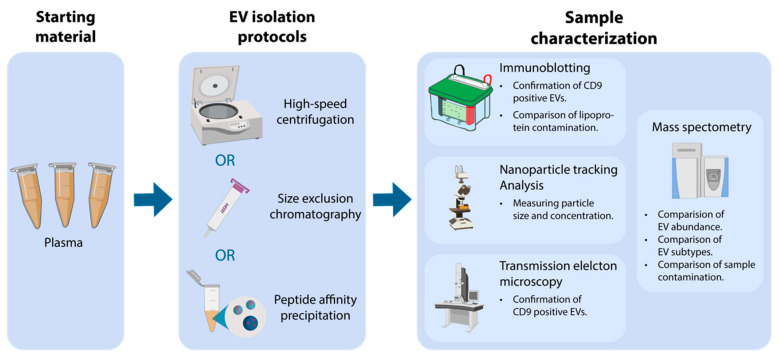
Overview of the applied workflow starting with plasma, isolation of extracellular vesicles (EV), followed by sample characterization. EVs were isolated using either high-speed centrifugation, size exclusion chromatography (SEC), or peptide affinity precipitation (PAP). EV isolates were characterized by nanoparticle tracking analysis (NTA), transmission electron microscopy (TEM), immunoblotting, and mass spectrometry (MS).

In this work, we employ a comprehensive workflow (Figure 1) to evaluate and compare three EV isolation protocols including high-speed centrifugation, SEC, and PAP. First, to confirm EV isolation, we evaluated particle concentration, particle mode size, and CD9 presence. Secondly, we characterized the EV proteome and estimated EV abundance, differences in EV subtypes (large and small EVs), sample contamination (serum albumin, lipoproteins, and non-EV proteins), and reproducibility of each isolation protocol.

### 3.1. Isolation of Extracellular Vesicles

**Figure 2:**
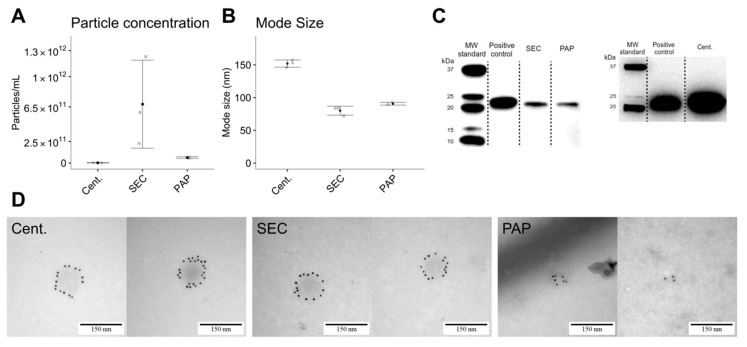
EV characterization by nanoparticle tracking analysis (NTA), immunoblotting, and transmission electron microscopy (TEM). (**A**) Differences in particle concentration measured by NTA. (**B**) Differences in particle mode size measured by NTA. (**C**) Immunoblot against CD9 (24 kDa) confirming the presence of the EV specific protein CD9 in isolates from all methods. (**D**) TEM images showing CD9 expression in vesicle-like particles in all three isolation methods. Error bars: Mean ± SD.

EVs were present in isolates from all isolation protocols, evidenced by NTA, TEM, and immunoblotting. Structural characterization by NTA showed that isolates produced by high-speed centrifugation contained larger particles (153 nm ± 6 nm) with a relatively low concentration (3.69 × 10^9^ ± 1.13 × 10^9^ particles/mL). SEC isolates had the smallest particles (80 nm ± 7 nm) and highest concentration (6.81 × 10^11^ ± 5.08 × 10^11^ particles/mL). Isolates produced by PAP had particles of comparable size to SEC isolated particles (91 nm ± 5 nm) and a particle concentration of 6.40 × 10^10^ ± 9.32 × 10^9^ particles/mL (Figure 2A,B). Size distribution profiles from the NTA is avaiable in Figure S1 and Table S3. Phenotype characterization by immunoblotting and TEM confirmed the presence of CD9 positive EVs in all isolates (Figure 2C,D).

### 3.2. Unique Proteomes

**Figure 3:**
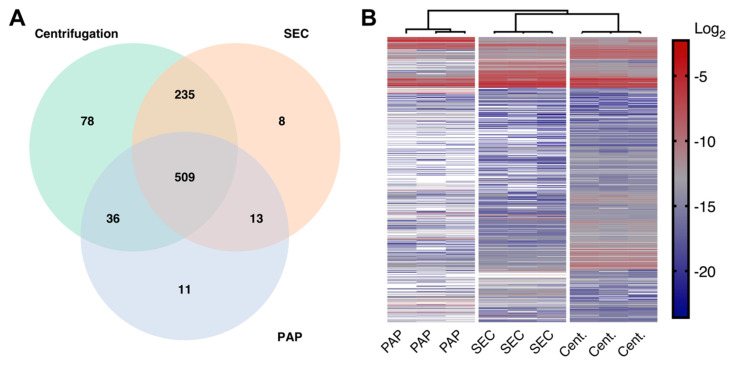
Differences in the EV proteomes in isolates produced by high-speed centrifugation (Cent.), size exclusion chromatography (SEC), and peptide affinity precipitation (PAP). (**A**) Venn diagram showing common and unique proteins identified in all three methods. (**B**) Hierarchical clustering of protein intensities from isolates obtained by high-speed centrifugation, SEC, and PAP showing that the three isolation methods resulted in three distinct EV proteomes. White = missing values.

Mass spectrometry-based proteome analysis revealed that the evaluated protocols resulted in three distinct EV proteomes of varying complexities. The EV isolate produced by high-speed centrifugation showed the most complex proteome with a total of 858 proteins. In contrast, SEC and PAP isolates were less complex with 765 and 569 proteins, respectively (Figure 3A). In general, the protein overlap between methods was high with 509 (57%) proteins in common. When comparing protein abundance profiles by hierarchical clustering (settings; Euclidean distance; average linkage), samples obtained by the three isolation methods clustered separately, again indicating unique protein profiles for each EV isolate (Figure 3B).

### 3.3. Quantitative Differences in Extracellular Vesicle Proteome

**Figure 4:**
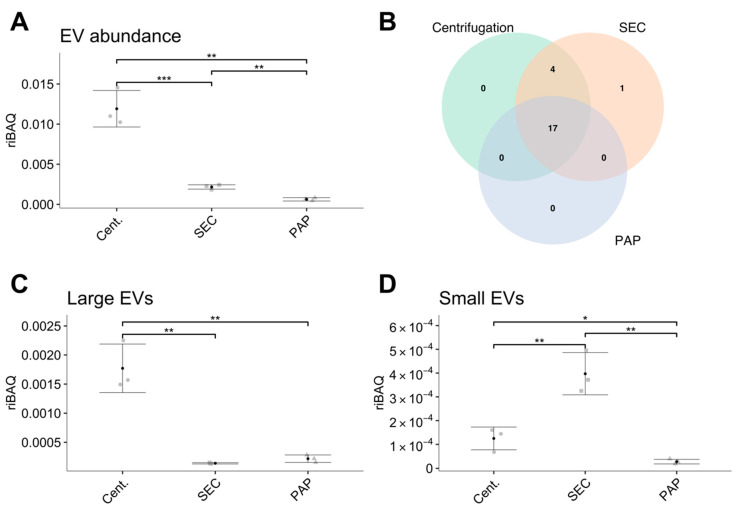
Differences in the abundance and amount of extracellular vesicle (EV) specific proteins in isolates obtained by high-speed centrifugation (Cent.), size exclusion chromatography (SEC), and peptide affinity precipitation (PAP). (**A**) The difference in abundance of EV specific proteins. (**B**) Venn diagram showing the number of identified EV specific proteins per isolation method. (**C**,**D**) Comparison of relative abundances of EV subtypes divided into large EVs (**C**) and small EVs (**D**). Error bars: Mean ± SD. Significance levels: * (*p*-value < 0.05), ** (*p*-value < 0.01), and *** (*p*-value < 0.001).

Relative EV abundance was estimated by summing riBAQ values for EV associated proteins, as described in materials and methods. By this calculation, isolates obtained by high-speed centrifugation had the highest EV abundance, SEC isolates had medium EV abundance while PAP isolates had the lowest EV abundance (Figure 4A). In relative terms, high-speed centrifugation had a 5.5-fold higher EV abundance compared to SEC isolates (*p* < 0.001), and a 19.0-fold increased EV abundance compared to PAP isolates (*p* = 0.005). SEC isolates had 3.5-fold higher EV abundance (*p* = 0.008) compared to PAP isolates. When comparing the number of identified EV specific proteins, high-speed centrifugation isolates contained 21, SEC isolates contained 22, while PAP isolates contained 17 EV specific proteins (Figure 4B).

When focusing on differences in EV subtypes, we found that all protocols resulted in the isolation of proteins associated with both large and small EVs. High-speed centrifugation isolates were significantly enriched in proteins specific to large EVs (Figure 4C), while SEC isolates had significantly higher amounts of proteins specific to small EVs (Figure 4D). PAP isolates contained a low amount of both large and small EV specific proteins.

### 3.4. Co-isolation of Contaminants

**Figure 5:**
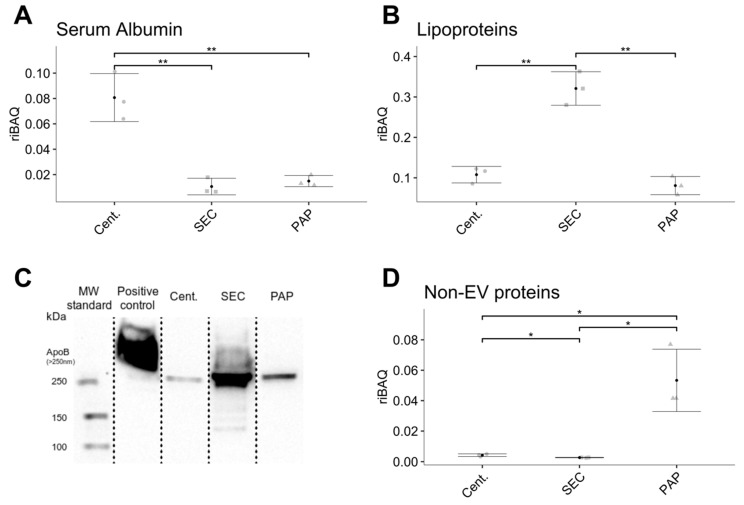
Difference in the co-isolation of contaminants such as serum albumin, lipoproteins, and other non-EV proteins between isolates produced by high-speed centrifugation (Cent.), size-exclusion chromatography (SEC), and peptide affinity precipitation (PAP). (**A**) High-speed centrifugation produced EV isolates with the highest abundance of serum albumin. (**B**) SEC isolates contained the highest amount of lipoproteins. (**C**) Immunoblotting confirms elevated levels of apolipoprotein B in isolates produced by SEC. (**D**) PAP isolates contained the highest amount of non-EV proteins. Error bars: Mean ± SD. Significance levels: * (*p*-value < 0.05) and ** (*p*-value < 0.01).

To evaluate sample purity, we assessed to which extent the different EV isolates co-isolated contaminating proteins. Contaminating proteins were defined as serum albumin, lipoproteins, and other non-EV proteins (Table S2). Comparing these proteins, we found that high-speed centrifugation isolates contained a significantly higher amount of serum albumin compared to the other methods (Figure 5A) and that SEC derived isolates showed a higher abundance of lipoproteins (Figure 5B). EVs isolated by PAP resulted in a low abundance of both serum albumin and lipoproteins. Quantitatively, high-speed centrifugation had a 7.6-fold higher abundance of serum albumin compared to SEC (*p* = 0.003) and a 5.4-fold higher abundance compared to PAP isolates (*p* = 0.008). Focussing on lipoproteins, SEC showed a 3.0-fold and a 4.0-fold higher abundance of lipoprotein compared to high-speed centrifugation (*p* = 0.001) and PAP isolates (*p* = 0.003), respectively. Immunoblotting against ApoB-48/100 confirmed that lipoproteins were present in higher amounts in isolates produced by SEC (Figure 5C).

In addition to evaluating the abundance of serum albumin and lipoproteins, we examined differences in non-EV associated proteins as a joint surrogate marker for the general degree of sample purity. The abundance of non-EV proteins was calculated as the sum of riBAQ values of all proteins not listed in Exocarta [30] (http://exocarta.org/Archive/EXOCARTA_PROTEIN_MRNA_DETAILS_5.txt) and included 66 proteins (Table S1). This comparison revealed that the PAP protocol resulted in isolates with the highest abundance of non-EV proteins; isolates from high-speed centrifugation showed a medium abundance of non-EV proteins, while SEC isolates showed the lowest abundance (Figure 5D). Quantitatively, PAP isolates had 19.8-fold more non-EV proteins compared to SEC (*p* = 0.02) and 12.5-fold more compared to high-speed centrifugation isolates (*p* = 0.01). High-speed centrifugation isolates had 1.6-fold more non-EV proteins compared to SEC isolates (*p* = 0.01).

### 3.5. Reproducibility of Isolation Methods

**Figure 6:**
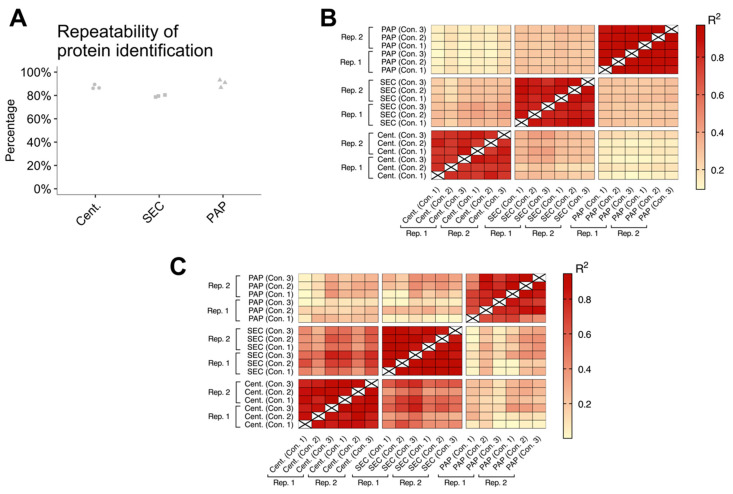
Reproducibility of the extracellular vesicle (EV) isolation methods. Reproducibility was evaluated for each biological sample by technical replicates and was evaluated for protein identifications repeatability (**A**) and correlation matrix of all protein abundances (**B**+**C**). (**A**) Proteins identified in all technical replicates of EV samples isolated by using the same method. (**B**+**C**) Correlation matrix (coefficient of determination (*R^2^*)) of the reproducibility between technical replicates (Rep. 1 and Rep. 2) and biological controls (Con. 1, Con. 2, and Con. 3) of the evaluated EV isolation protocols.

For an EV isolation method to be applicable in clinical proteomic biomarker studies, the method must produce EV isolates with high reproducibility. Reproducibility was evaluated for each biological sample using technical replicates. Reproducibility was assessed by calculating the repeatability of protein identifications and coefficient of determination (R^2^) of protein abundances (riBAQ) between the technical replicates. The repeatability of protein identifications varied between 78% and 93%, where PAP had the highest repeatability (mean: 90%; range: 87–93%), followed by high-speed centrifugation (mean: 87%; range: 86–89%), and SEC (mean: 79%; range: 79–80%), (Figure 6A). Evaluating the coefficient of determination of protein abundances, we found a high similiariy between the technical replicates, with a coefficient of determination in the range from 0.75 to 0.97 (Figure 6B). No similarity was observed between EV isolates from different isolation methods. When evaluating the coefficient of determination using only EV specific proteins (Figure 6C) EV isolates produced by high-speed centrifugation and SEC had a high similarity of their respective technical replicates (R^2^: 0.79–0.94). Furthermore, EV isolates produced by high-speed centrifugation had some similarity to the EV isolates produced by SEC (R^2^: 0.27–0.81). The coefficient of determination for EV isolates produced by PAP was more varying (R^2^: 0.41 to 0.93).

## 4. Discussion

EV isolation is challenging and is complicated by the size overlap between different EV subtypes and their heterogeneous distribution within most tissues and body fluids [9,14,19]. Using phenotypical and structural evaluation methods recommended by ISEV [32], we confirmed that all evaluated isolation protocols were capable of isolating EVs. Furthermore, we found that the three methods all had high reproducibility, both concerning protein identifications and protein abundances. However, PAP showed lower reproducibility when focusing on EV specific proteins alone. Combined, these results show that high-speed centrifugation and SEC can consistently isolate EVs, while PAP is more varying in its performance.

Even though all methods successfully isolated EVs, there were considerable differences in the resulting proteomes. The EV isolate produced by high-speed centrifugation had the most complex proteome, followed by the isolate produced by SEC. Isolates produced by PAP showed the lowest complexity. These differences were expected and consistent with the differences in the actual mechanisms of isolation [14,15,19,33].

Addressing these proteome differences in relation to EV abundance, we found that the EV isolates produced by high-speed centrifugation resulted in substantially higher EV abundance compared to SEC and PAP. We did however also find a similar number of EV specific proteins in high-speed centrifugation compared to SEC, implying that SEC contains a similar EV population, however, at a lower relative amount. Nevertheless, compared to PAP, both SEC and high-speed centrifugation isolates showed higher EV abundance and more EVs specific proteins. In line with the differences in EV subtype, our data clearly shows that high-speed centrifugation isolates contain the highest amount of proteins associated with larger EVs while SEC contains the highest amount of proteins associated with smaller EVs. Combined, these results are in accordance with current knowledge, where high-speed centrifugation is expected to isolate primarily MVs and SEC expected to isolate a combination of MVs and exosomes [18]. PAP isolates showed a low abundance of both large and small EV specific proteins.

Our finding of a general low EV content in isolates produced by PAP using the ME kit is contradictory to Knol et al. [14], who reported that the ME kit resulted in similar EV abundance as with centrifugation based methods. Since the Vn96-peptide applied in the ME-kit is designed to specifically bind to HSP70, which is proposed upregulated on EVs during cellular stress, our use of healthy controls could have negatively affected EV yield [34]. Nevertheless, our results do indicate that PAP is suboptimal for isolation of EVs from healthy controls in plasma.

A common challenge with EV isolation is the co-isolation of non-EV proteins [9,18]. This challenge is especially prominent in blood derivatives such as plasma, due to its high complexity and dynamic range [3]. Obtaining an EV isolate of high purity in biomarker studies is important, as non-EV proteins can potentially suppress EV proteins and complicate the evaluation of whether a property is truly associated with EVs [3,35]. Our approach by quantifying non-EV markers and other proteins not listed in Exocarta showed that high-speed centrifugation and PAP based isolates show the highest relative abundance of non-EV contaminants, indicating lower sample purity. The low purity of EV isolates produced by high-speed centrifugation is most likely a consequence of the method’s non-specific mechanism of isolation, where contaminants of similar density and size will co-isolate with EVs and the simple fact that contaminants close to the bottom of a tube will co-isolate with the EV pellet due to their relatively short segmentation distance. Nevertheless, a limitation with evaluating non-EV proteins using this approach is that it does not take highly abundant proteins such as serum albumin or lipoproteins into consideration, as these are included in the Exocarta database. Hence, these factors were compared independently.

The co-isolation of serum albumin is unavoidable when isolating EVs from plasma, as serum albumin is the most abundant plasma protein and accounts for approximately half of the total plasma protein content. Serum albumin can complicate EV isolation by high-speed centrifugation by the formation of protein aggregates with similar size and density as EVs. The amount of serum albumin in EV isolates from high-speed centrifugation can be reduced by repeated centrifugation but at the cost of a lower EV yield for each centrifugation step. Five high-speed centrifugation steps with four pellet washes seem to be optimal for serum albumin removal without a considerable decrease in EV yield [10]. In comparison, SEC isolated EVs had minimal serum albumin content, which is in line with the findings of Baranyai and colleagues [18], where levels of serum albumin were significantly higher in EV isolates prepared by ultracentrifugation compared to SEC.

Focusing on lipoproteins, we showed that isolates produced by SEC had approximately three times as many lipoproteins than isolates obtained by high-speed centrifugation and PAP. Lipoproteins accounted for approximately 30% of the total protein content in SEC based isolates. Besides the impact on EV purity, the presence of lipoproteins is also problematic because chylomicrons and very-low-density lipoproteins are in the same size-range as EVs [36]. This means that co-isolated chylomicrons (75–1200 nm) and very-low-density lipoproteins (30–80 nm) will be counted as EVs during NTA characterization, confounding NTA results [17,37]. Contrary to our findings, reports from Böing and colleagues claim that EV isolation by SEC leads to almost complete removal of lipoproteins [16]. However, this conclusion was based solely on the presence of high-density lipoprotein, which only accounts for a fraction of all lipoproteins, and has minimal size overlap with EVs [16]. Isolates obtained by high-speed centrifugation and PAP showed minimal lipoprotein contamination. Nevertheless, all EV isolates produced still had considerable contamination. Combined, contaminating proteins accounted for ~10–30% of the total protein content depending on the isolation method. This apparent limitation implies that there is still a need for improved isolation procedures. One strategy which has recently gained popularity is the combination of several methodologies, such as a combination of centrifugation and SEC [32]. While such methods can improve EV purity, they also add complexity to the sample preparation procedures, such as potential reduced EV yield [38], and being more labour intensive. Hence, EV isolation procedures must have a fine balance between performance and applicability.

The main limitation of this study is the low sample size consisting of three individuals. As such, there is a risk that the presented data have low statistical power. Nevertheless, our study does not attempt to define potential biomarkers, but more broadly characterise proteome differences between EV isolates. Furthermore, many of the identified are large, mitigating the effect of having lower statistical power.

## 5. Conclusions

Despite great interest in EVs and their associated proteins, there is currently no consensus on the preferred method for EV isolation in MS-based biomarker studies. In this work, we evaluated and compared three different EV isolation protocols for their applicability in MS proteome-based biomarker research. Based on the presented work, our main finding is that both high-speed centrifugation and SEC are suitable for EV isolation for MS-based biomarker discovery, but that they favor large and small EVs respectively, and thereby result in different proteomes. The challenge with sample purity may not pose a problem for all downstream applications, but its importance should always be considered. EV isolates obtained by PAP had low EV content and considerable co-isolation of non-EV components, making the method less suited for MS-based proteome biomarker studies. The choice between the SEC and high-speed centrifugation should depend on the scientific questions and whether the focus is on smaller EVs, larger EVs, or a combination of both.

## Supplementary Materials

Supplementary materials can be found at https://www.mdpi.com/2227-9059/8/8/246/s1, Table S1: Processed_MS_data.txt, Table S2: EV_and_contamination_markers.ods, Table S3: NTA_data.ods, Figure S1: NTA_size_distribution.png, File S1: Readme.pdf, File S2: Undedited_images, File S3: R_code, File S4: Perseus_analysis_file.sps, File S5: Raw MaxQuant data, File S6: Exported Perseus data.
