# Supplementary material for: Mass-Spectrometry Based Proteome Comparison of Extracellular Vesicle Isolation Methods: Comparison of ME-kit, Size-Exclusion Chromatography, and High-Speed Centrifugation
Source: Biomedicines. 2020 Jul 25;8(8):246. doi: 10.3390/biomedicines8080246 (PMC7459681; doi:10.3390/biomedicines8080246)
Supplement: Supplementary file 1 [file biomedicines-08-00246-s001.zip › File S1 - Readme.pdf]

File S1 - Readme

This file is intended to give an overview of all supplementary material provided with this manuscript.

Supplementary Tables

1. Table S1 - Processed_MS_data.txt

- This file contains the processed MS data, where the data is filtered, have calculated riBAQ values, and is annotated.

2. Table S2 - EV_and_contamination_markers.ods

- This file contains the Gene ID for the different proteins which we have grouped and annotated our dataset based on. It includes: 1 Non-EV proteins (dataset 5 from exocarta), 2) Serum albumin, 3) Lipoproteins, 4) Total EV markers, 5) small EV markers, and 6) large EV markers.

3. Table S3 - NTA_data.ods

- This file includes the mean size, mode size, and concentration of EVs. Furthermore, it contains the raw data used to create size distribution graphs (figure 1S).

Supplementary Figures

1. Figure S1 - NTA_size_distribution.png

- Figure that shows the size distribution of NTA data.

Supplementary Files

1. File S1 - Readme.pdf

- This file.

2. File S2 - Unedited_images

- Contains all images and figures used for the manuscript in their unedited form.

3. File S3 - R_code

- Statistical analysis and figures.R
  - This file is the main data handling file for the study. It includes data import and tidying of NTA data, exploratory data analysis of NTA data, and generation of all figures and calculation of p-values for protein MS data.
- image_editing.R
  - This file creates some of the images used in the article. They are created from raw images.

- Correlation matrix.R
  - This file is intended to transform (pivot wide) the Perseus correlation data frame into a more readable format. It is used for figure 6b, and 6c.
- size_distribution.R
  - This file calculates the size distribution to make figure 1s.
- NTA-Data_import.R
  - This file imports and tidy NTA data. To be used for figure 1 and section about particle size and concentration.
- Functions.R
  - This file contains all custom functions and otherwise needed for data analysis.
- MS data processing.R
  - This file opens the MS (MaxQuant) data files, filter out contaminants, and calculates riBAQ values, and writes this to a new file.
- size_disribution.R
  - This file creates figure S1.

4. **File S4 - Perseus_analysis_file.sps**

- This is the Perseus analysis file used for the data analysis of protein data. The version used was Perseus 1.6.0.7.

5. **File S5 - Raw MaxQuant data**

- proteingroups.txt
  - This file is the raw MaxQuant proteingroup data. It contains a total of 21 samples. 15 samples (6 Cent, 6 SEC, and 3 PAP) is used for the comparison of the proteme, while the remaining 6 is used to evaluate reproducibility. The two sets are processed using "match-between runs" within each set.
- peptides.txt
  - This file is the raw MaxQuant peptides file.
- experimentalDesign.txt
  - This file is the raw MaxQuant experimentalDesign file.

6. **File S6 - Exported Perseus data**

- This folder contains all the exported Perseus data used for data analysis in R.
