# Supplementary figures and images for: Mass-Spectrometry Based Proteome Comparison of Extracellular Vesicle Isolation Methods: Comparison of ME-kit, Size-Exclusion Chromatography, and High-Speed Centrifugation
Source: Biomedicines. 2020 Jul 25;8(8):246. doi: 10.3390/biomedicines8080246 (PMC7459681; doi:10.3390/biomedicines8080246)

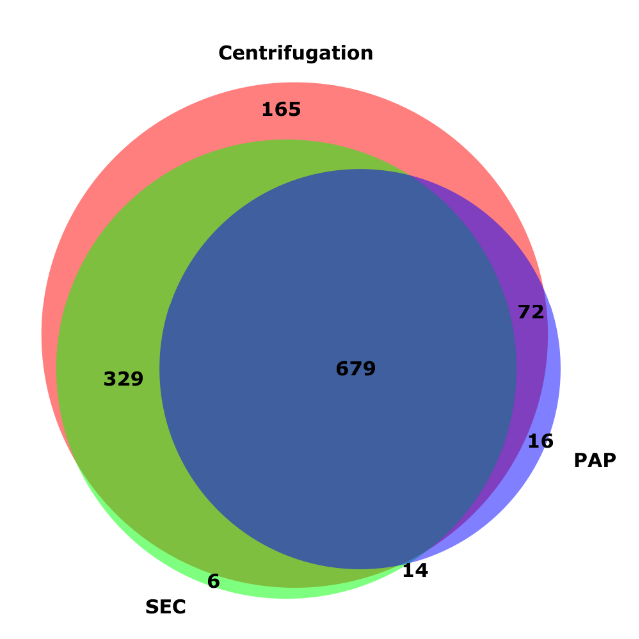

Supplement: Supplementary file 1 [file biomedicines-08-00246-s001.zip › File S2 - Unedited_images/Protein_venn_diagram.tiff]

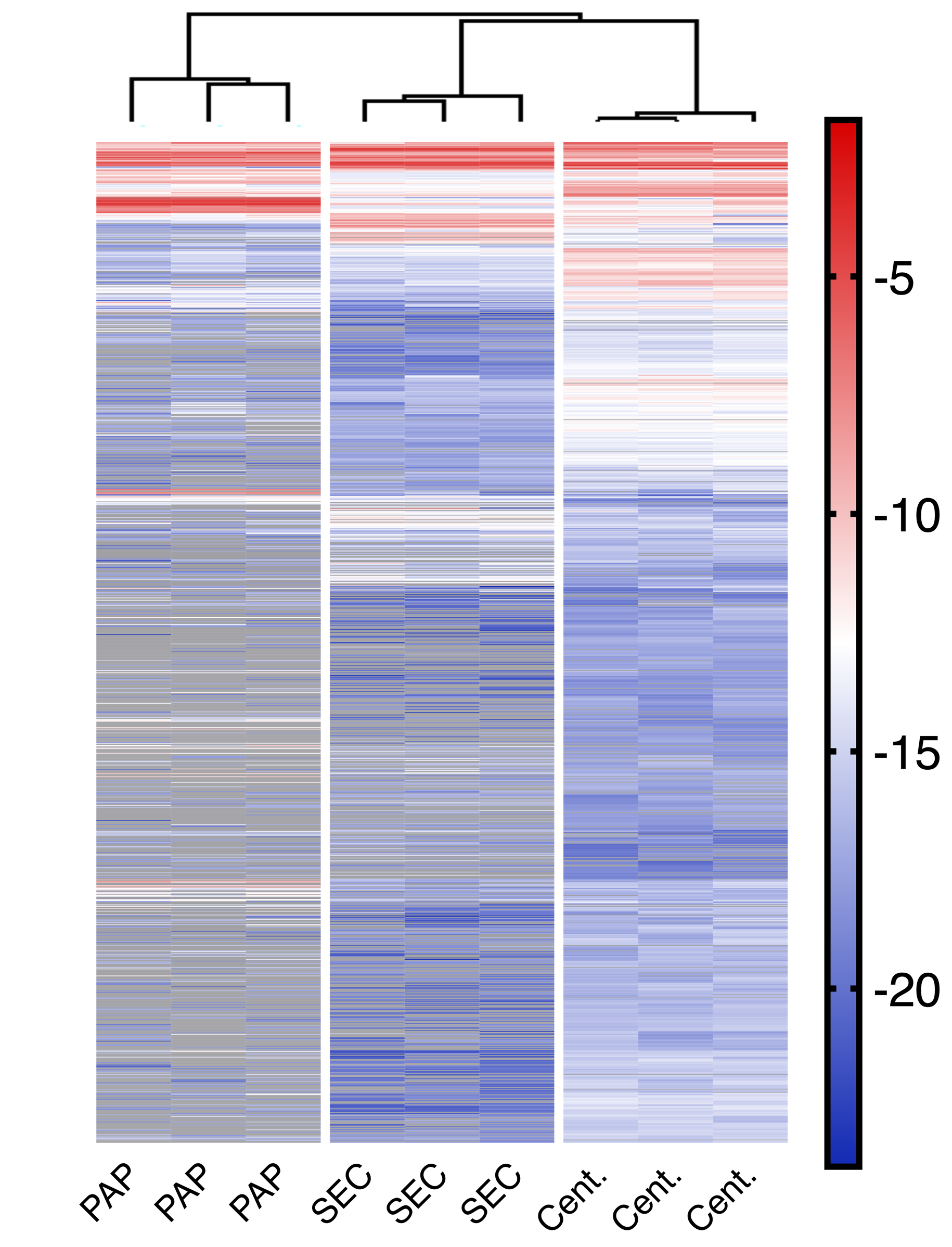

Supplement: Supplementary file 1 [file biomedicines-08-00246-s001.zip › File S2 - Unedited_images/Protein_clustering.png]

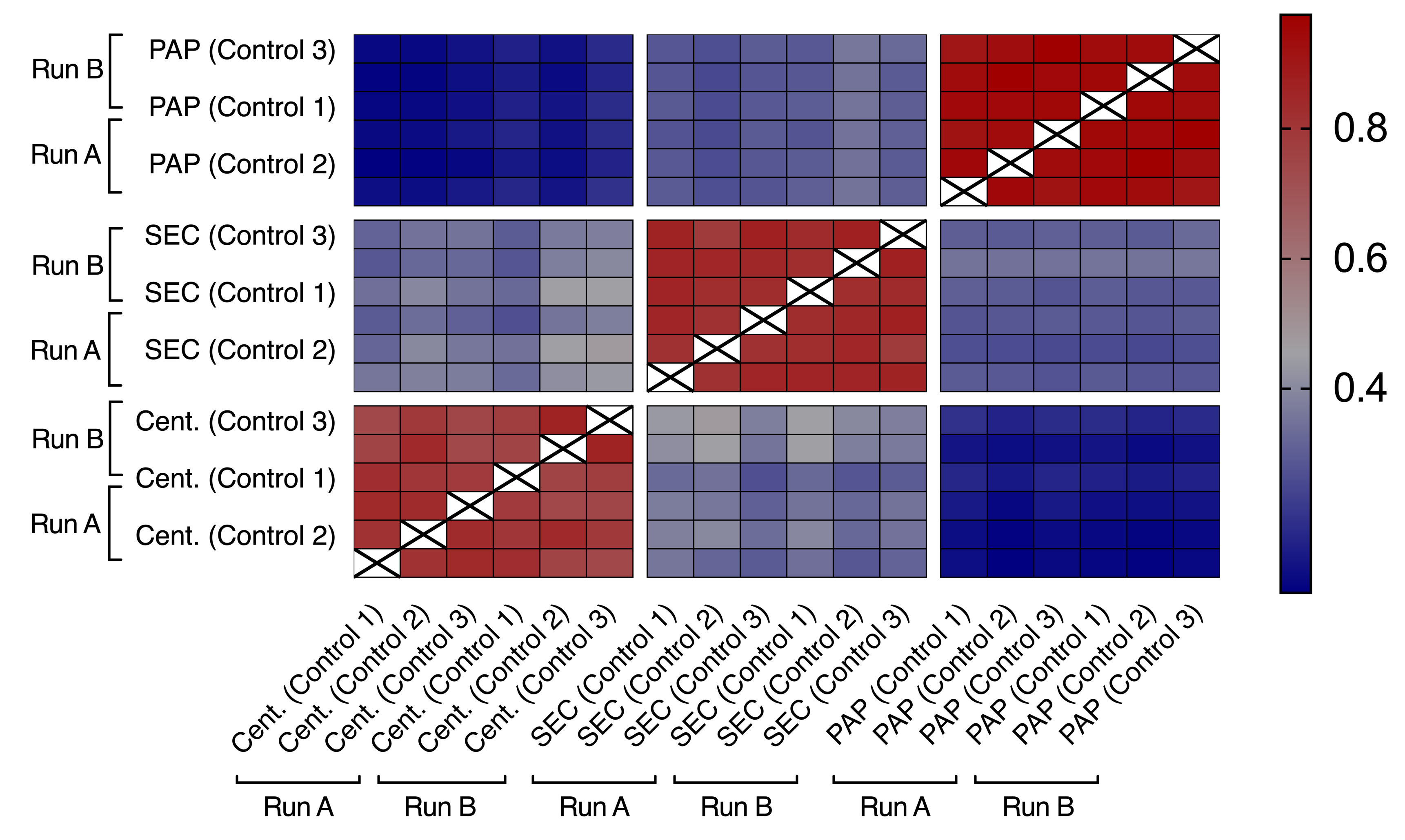

Supplement: Supplementary file 1 [file biomedicines-08-00246-s001.zip › File S2 - Unedited_images/Reproducability_correlation_matrix.png]

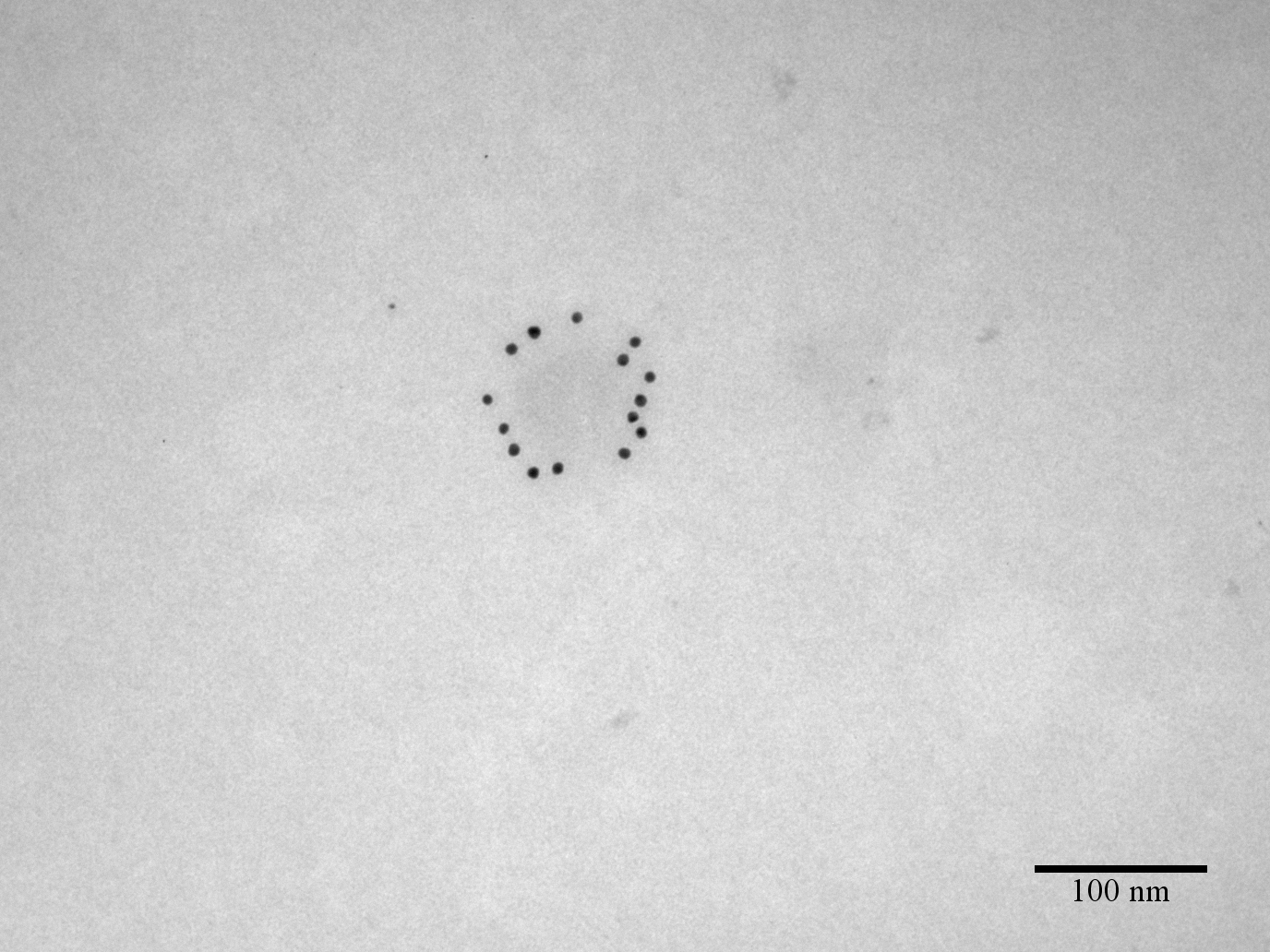

Supplement: Supplementary file 1 [file biomedicines-08-00246-s001.zip › File S2 - Unedited_images/TEM/SEC (2).png]

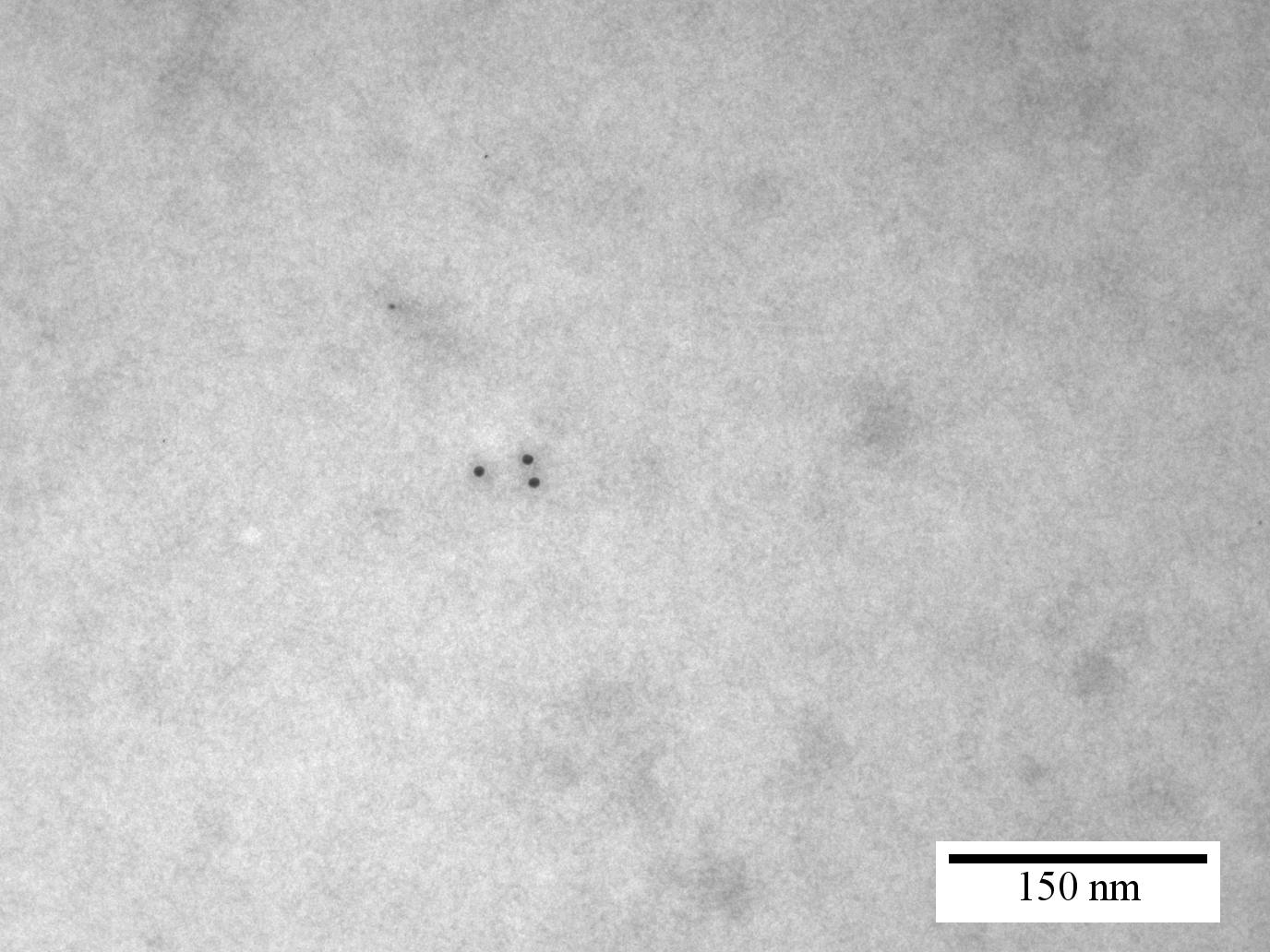

Supplement: Supplementary file 1 [file biomedicines-08-00246-s001.zip › File S2 - Unedited_images/TEM/scale_bar 2.png]

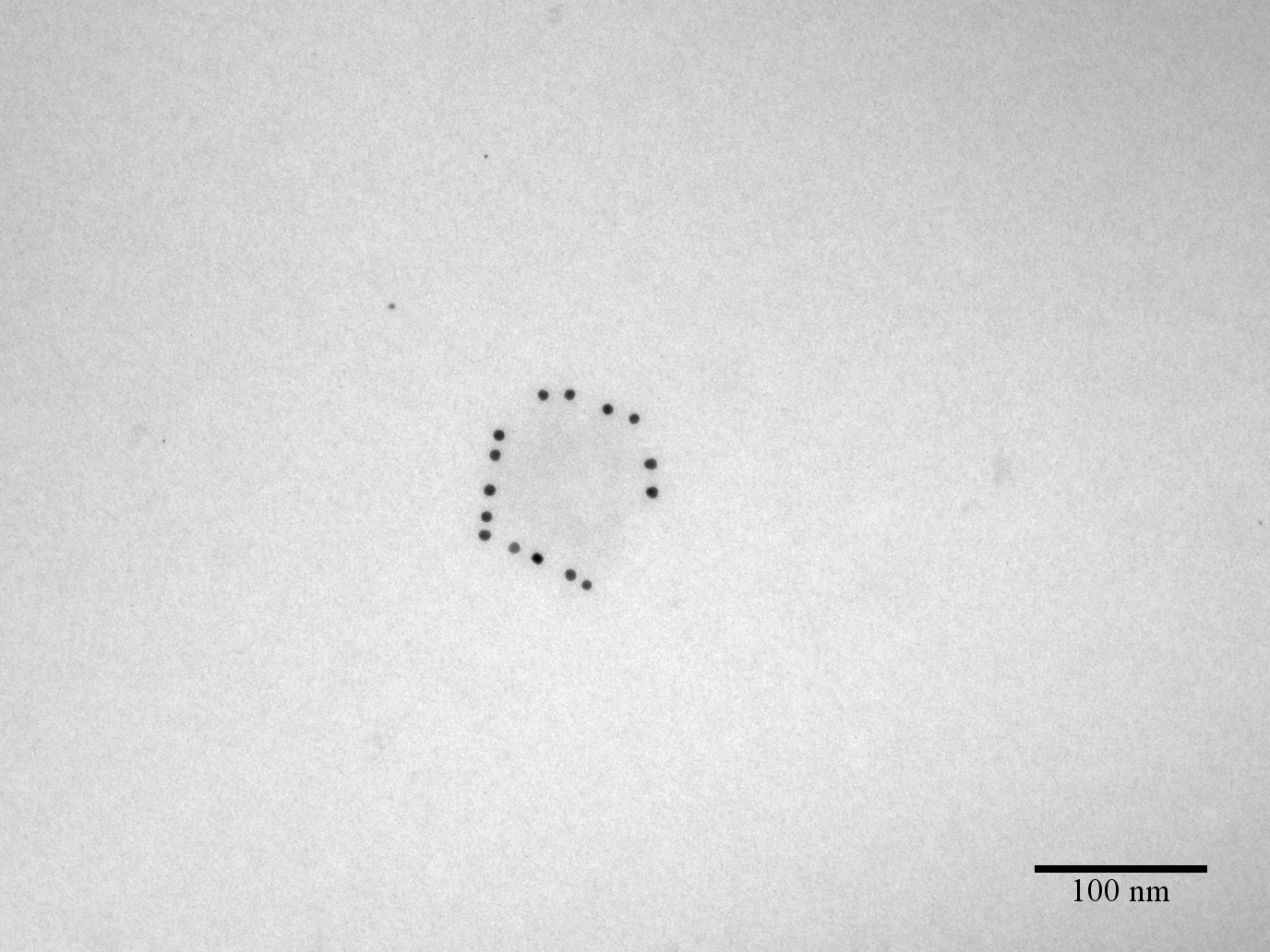

Supplement: Supplementary file 1 [file biomedicines-08-00246-s001.zip › File S2 - Unedited_images/TEM/Cent (1).png]

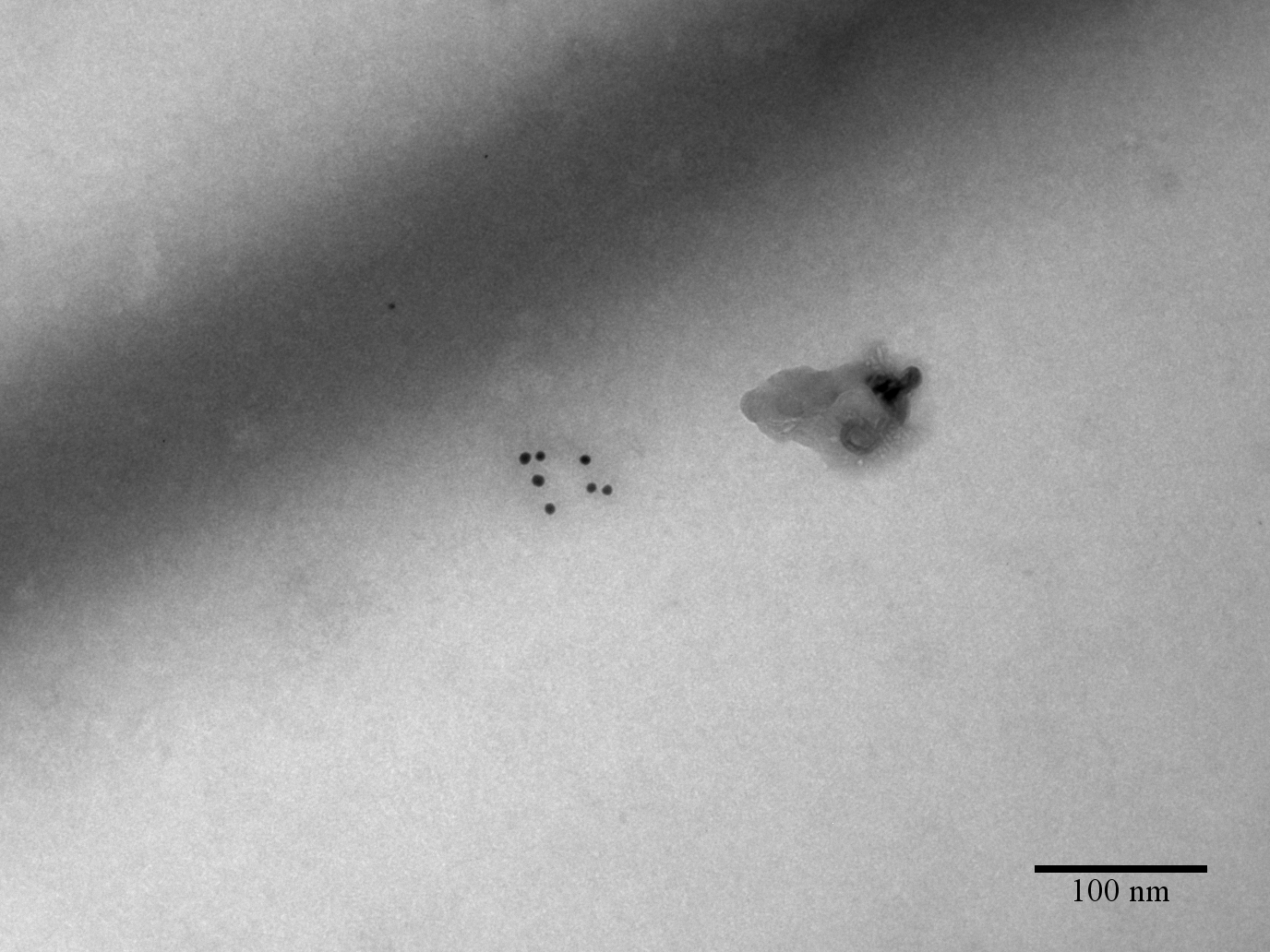

Supplement: Supplementary file 1 [file biomedicines-08-00246-s001.zip › File S2 - Unedited_images/TEM/Vn96(1).png]

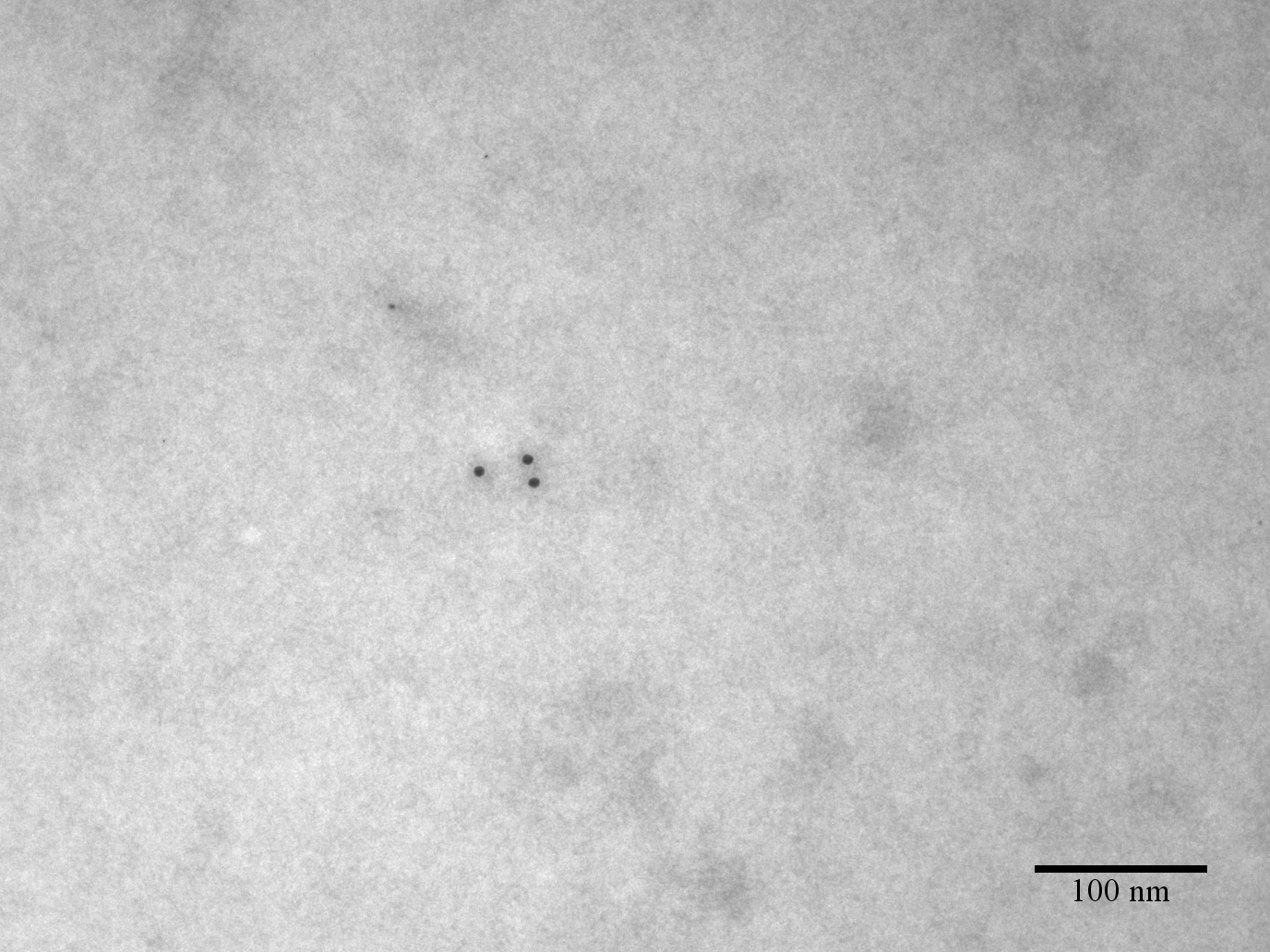

Supplement: Supplementary file 1 [file biomedicines-08-00246-s001.zip › File S2 - Unedited_images/TEM/Vn96(2).png]

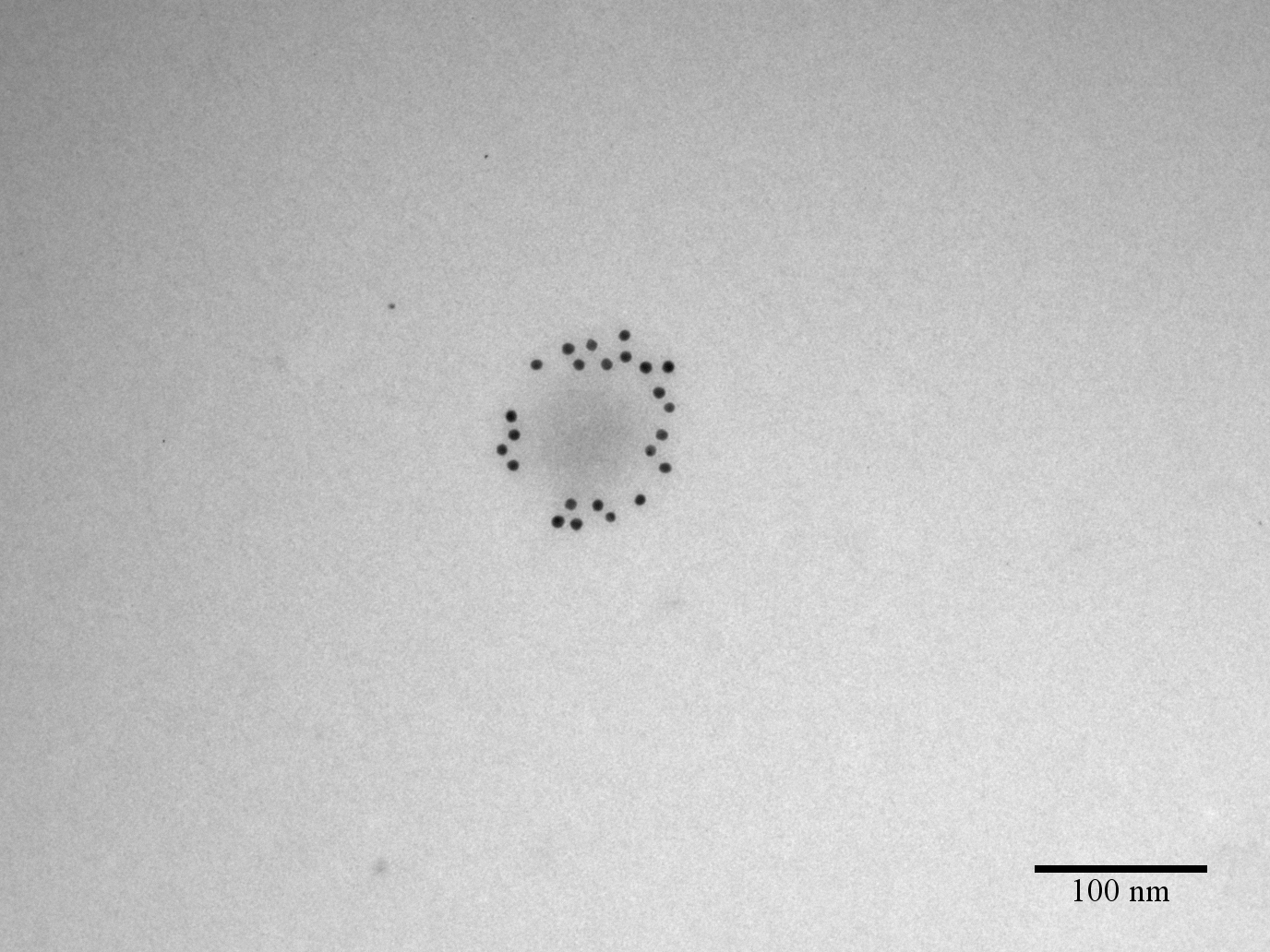

Supplement: Supplementary file 1 [file biomedicines-08-00246-s001.zip › File S2 - Unedited_images/TEM/Cent (2).png]

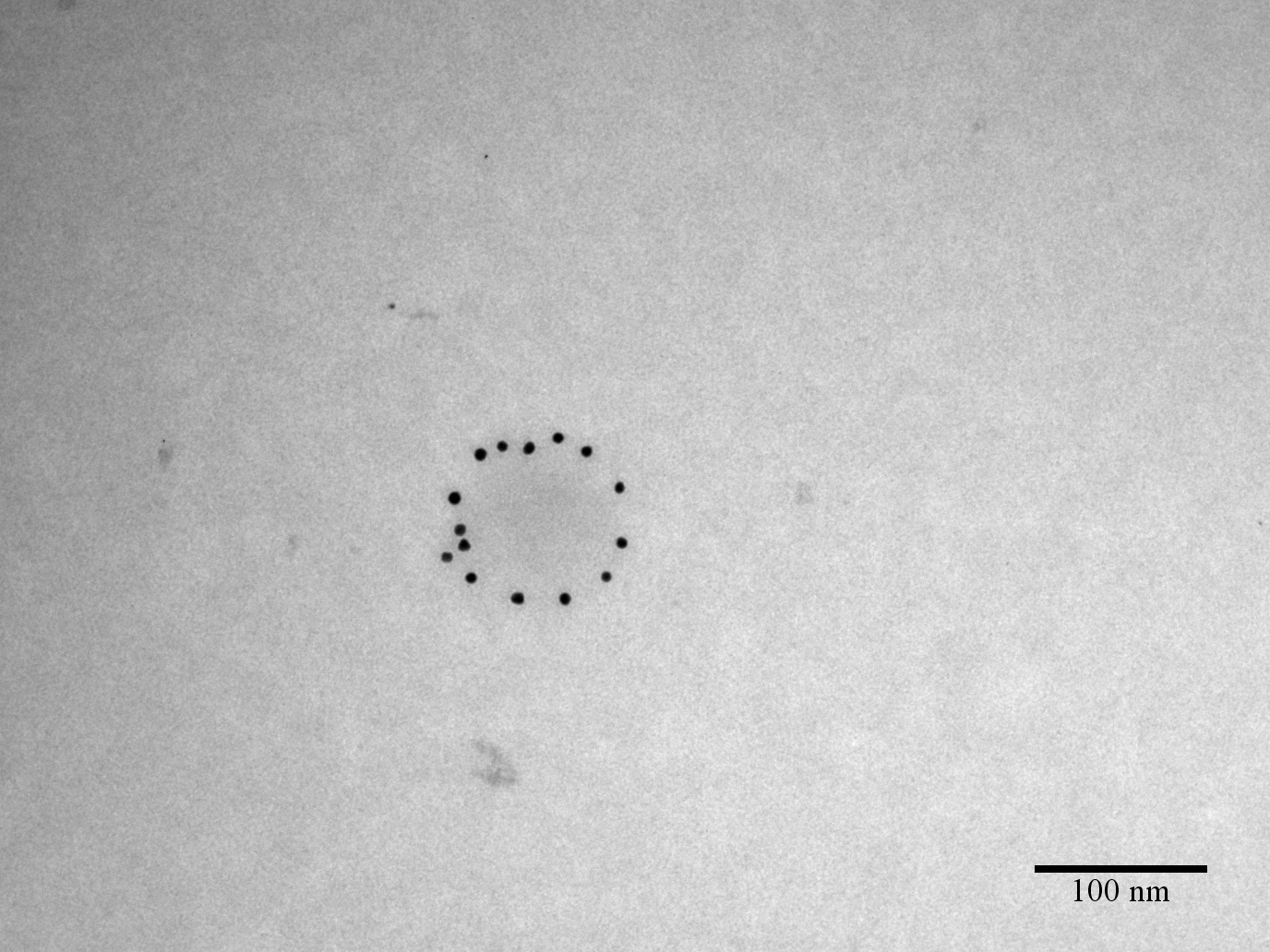

Supplement: Supplementary file 1 [file biomedicines-08-00246-s001.zip › File S2 - Unedited_images/TEM/SEC (1).png]

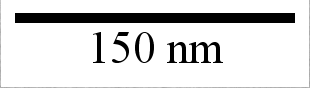

Supplement: Supplementary file 1 [file biomedicines-08-00246-s001.zip › File S2 - Unedited_images/TEM/scale_bar.png]

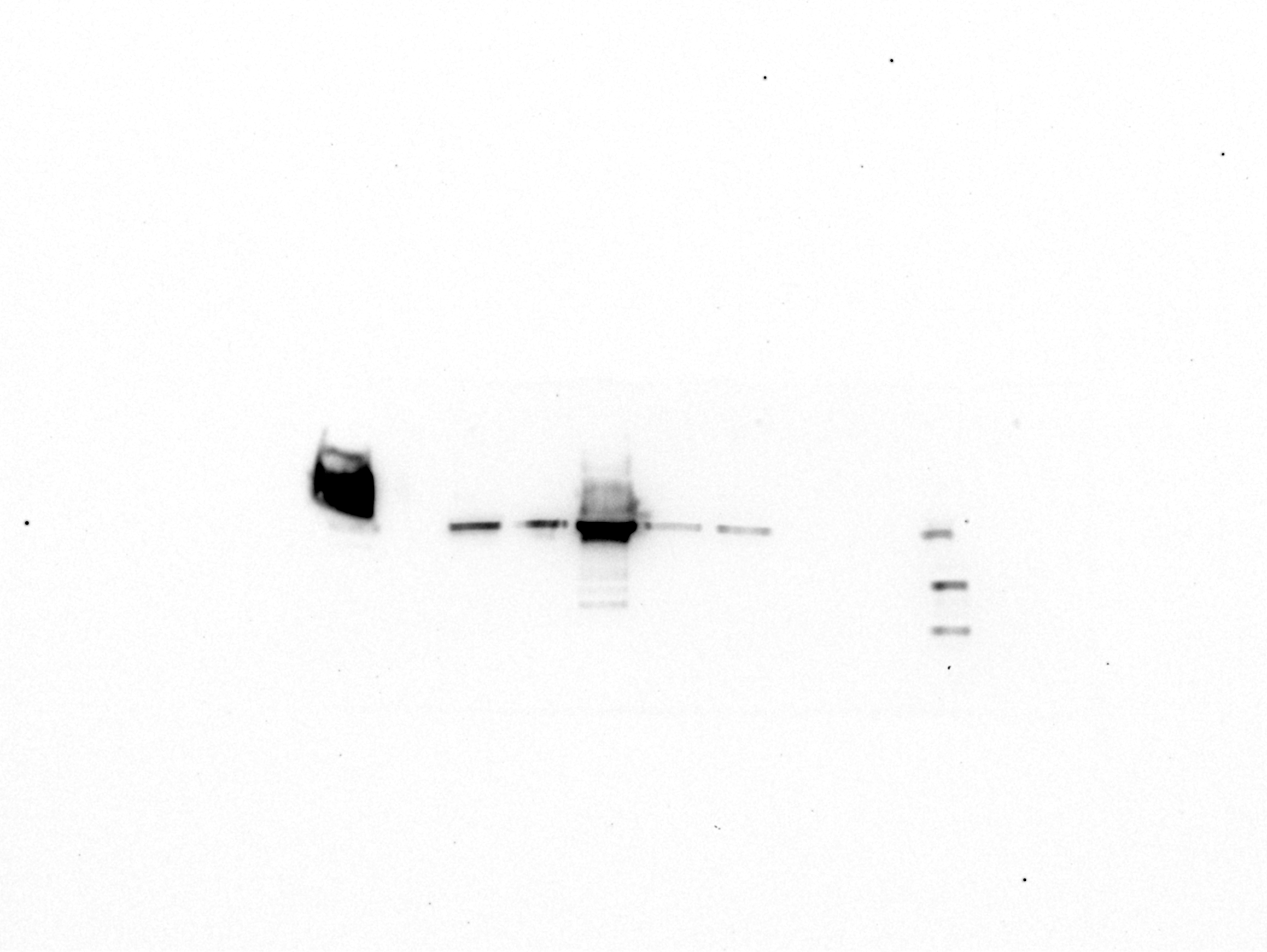

Supplement: Supplementary file 1 [file biomedicines-08-00246-s001.zip › File S2 - Unedited_images/Western blot/ApoB.tif]

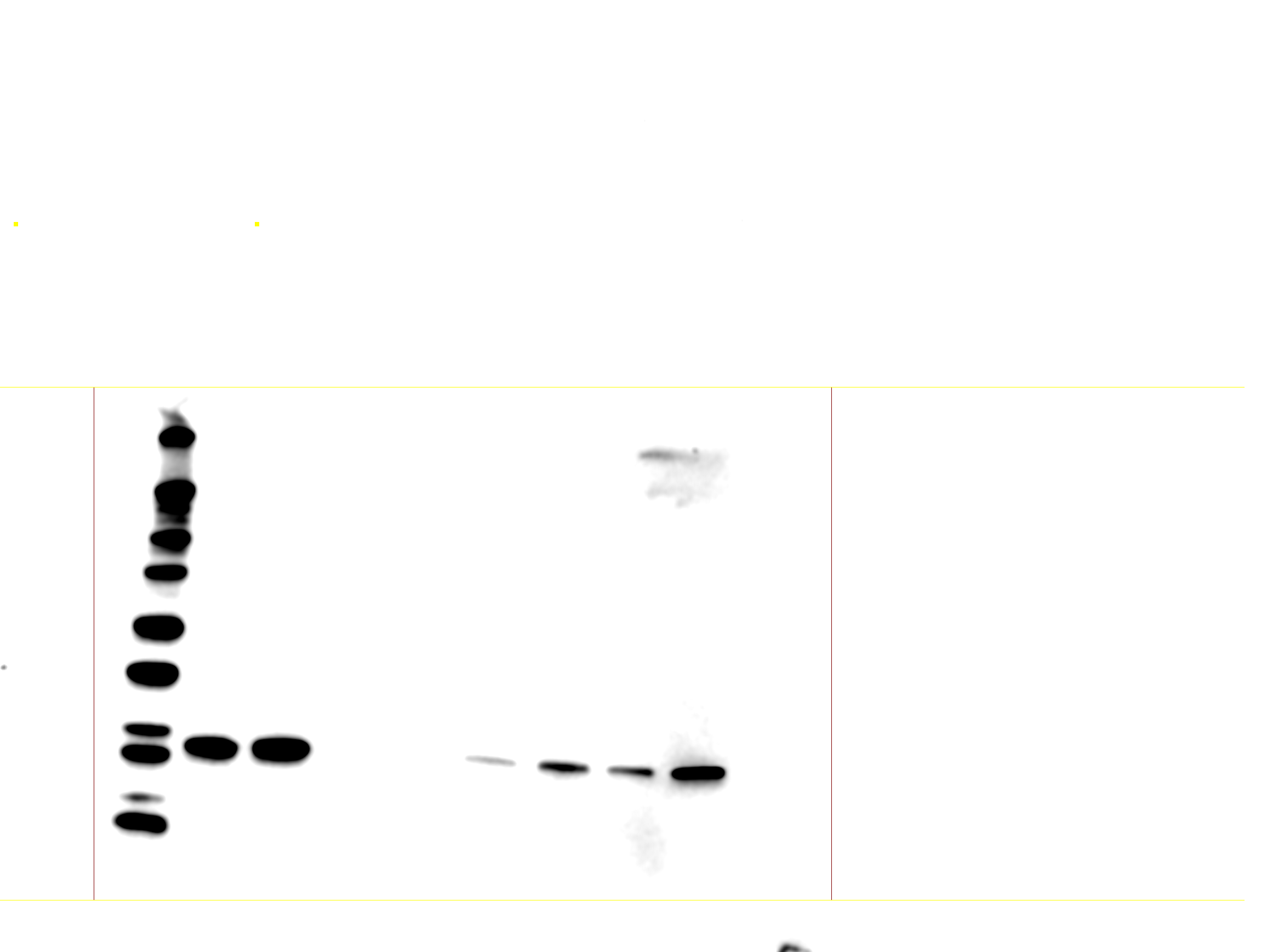

Supplement: Supplementary file 1 [file biomedicines-08-00246-s001.zip › File S2 - Unedited_images/Western blot/CD9.png]

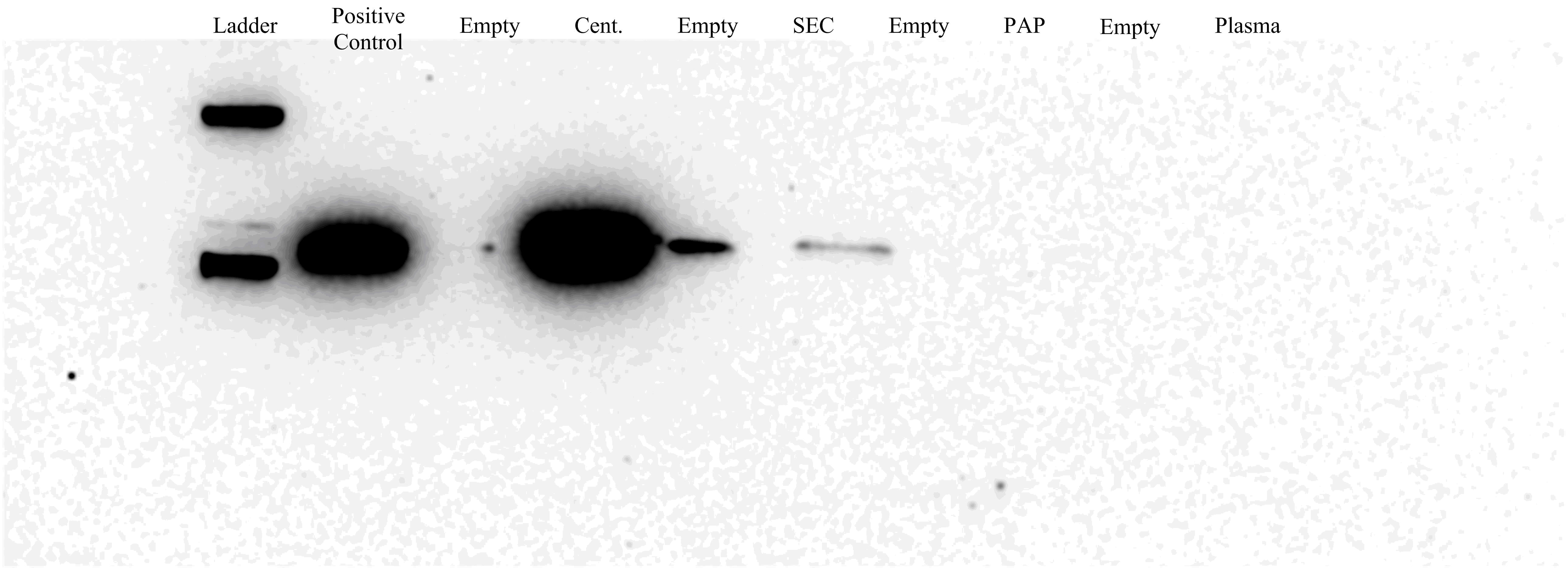

Supplement: Supplementary file 1 [file biomedicines-08-00246-s001.zip › File S2 - Unedited_images/Western blot/CD9_for_centrifugation.png]

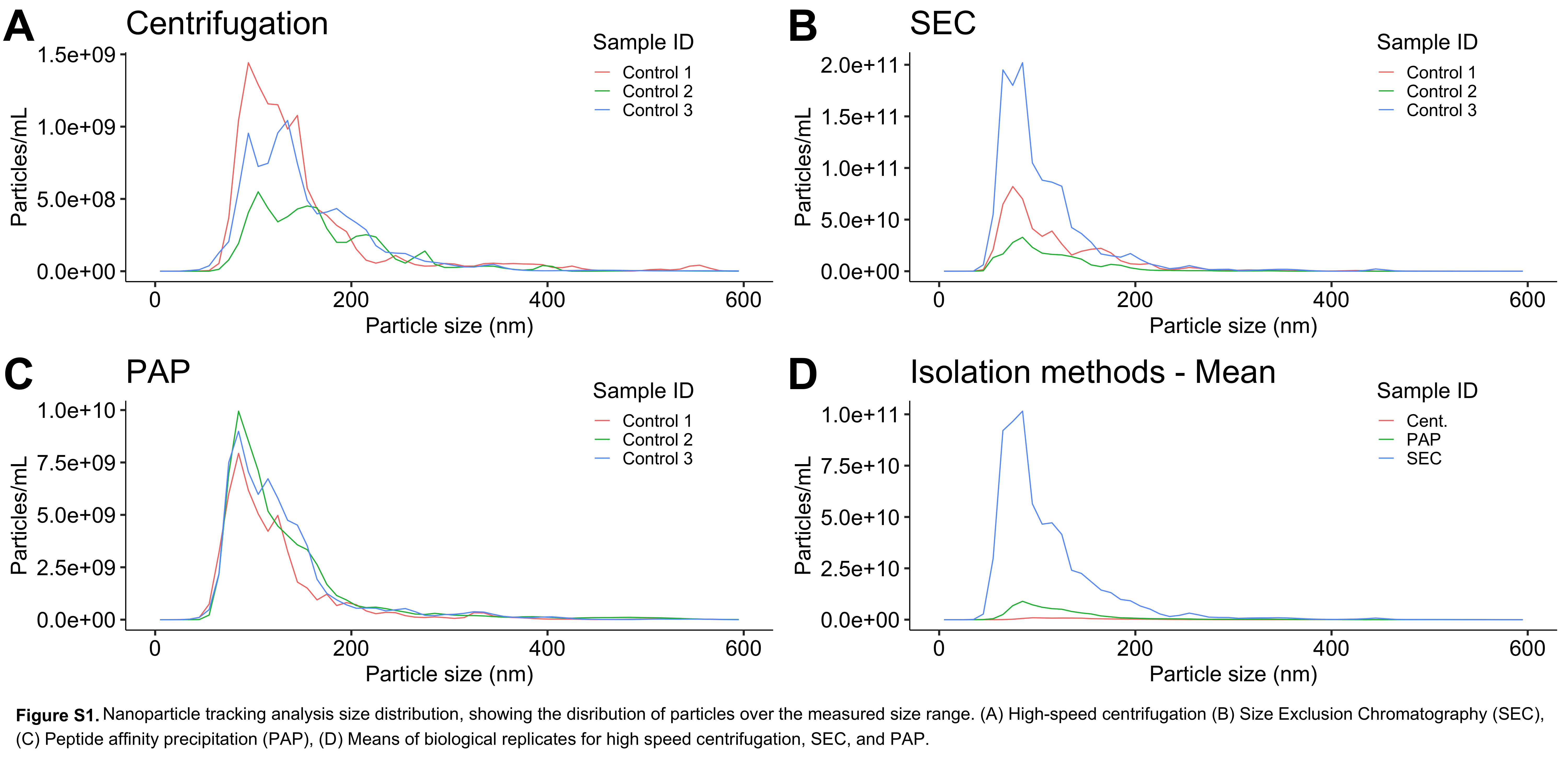

Supplement: Supplementary file 1 [file biomedicines-08-00246-s001.zip › Figure S1 - NTA_size_distribution.png]
